# Research on Influences of Transient High IOP during LASIK on Retinal Functions and Ultrastructure

**DOI:** 10.1155/2009/230528

**Published:** 2010-03-03

**Authors:** Haixia Zhao, Yude Ai, Chunmei Niu, Wenying Guan, Xiaoling Li, Liru Qin

**Affiliations:** Department of Ophthalmology, Affiliated Hospital of Inner Mongolia Medical College, Hohhot 010050, China

## Abstract

*Objectives*. To study the influences of transient high intraocular pressure(IOP) during LASIK on retinal functions and ultrastructure. *Methods*. Thirty-two New Zealand white rabbits were randomly divided into normal control, experimental control, negative suction 20 s and negative suction 3 min groups. The experimental control group was treated only by laser. Rabbit eyes received suction for different periods of time (20 s, 3 min) by negative pressure generator in different groups. The changes of neuro-optic and retinal ultrastructure were observed under electron and light microscopes; retinal neurofunctional changes were observed with flash-visual evoked potential (F-VEP) and flash-electroreinogram (F-ERG). *Results*. There was no obvious change in optic nerve, retina, ERG a-wave and b-wave in normal control and experimental control groups. There were slight changes in tissues of optic nerve and retina at various times of suction 20 s compared with control group, and a sharp change in suction 3 min group within 14d after operation, but these changes recovered at 28d . Amplitude of ERG b-wave observed at different time will decrease with suction periods prolonged. It can recover to normal level with the prolonged recovery periods. Amplitude and incubation period of ERG a-wave and VEP-P did not change significantly after different duration of suction. *Conclusions*. The transient high IOP during LASIK might have influence on retinal function and ultrastructure, but these changes were reversible.

## 1. Introduction

The security of LASIK is always a problem that people are concerned with. For example, whether the drastical rising and dropping of IOP evoked by negative suction during LASIK will lead to transient ischemia on retina and optic nerve, or whether the retinal structure and functions are further changing. According to previous studies, we have found the different changes of retinal ultrastructure [1] and retinal nerve fiber layer thickness [2] evoked by different negative suction times during LASIK. Clinically, we have also found that some people, after LASIK, appeared to have high IOP, and their nerve fiber layers were thinner than before [3]. Are these changes related to transient high IOP during LASIK? Will these changes further result in the changes of retinal function? Therefore, the aim this experiment is to explore the effect of negative suction during LASIK on retinal ultrastructure and retinal functions by using rabbits eyes as experimental models and to objectively evaluate the security time of negative suction during LASIK and the effect on retinal ultrastructure and retinal functions. 

## 2. Materials and Methods

### 2.1. Experimental Animals

Thirty-two healthy New Zealand white rabbits were offered by the Animal Laboratory of Inner Mongolia Medical College. Their average weight was nearly 4-5kg, both males and females, without eye disease.

### 2.2. Instruments and Reagents

Excimer laser (Shurong corporation, German); negative pressure generator (Moria II, France); optical microscope (Leica, German); microscopic photographic system (Olympus, Japan); JEM-1220 transmission electron microscope (electronics company, Japan); visual electrophysiology exam system (Medical device company of Chongqing Kang Hua, China).

### 2.3. Methods

#### 2.3.1. Grouping

New Zealand white rabbits were divided into normal control, experimental control, suction 20 seconds and negative suction 3 minutes groups, with 8 rabbits in each groups, their left eyes were used as experimental eyes. The changes of retinal ultrastructure were observed in postoperative instant, 7 days, 14 days and 28 days, respectively, and the changes of F-ERG(flash-electroretionogram) and F-VEP(flash-visual evoked potential) were observed in postoperative 10 minutes, 20 minutes, 30 minutes, 1 hour, 1 day, 7 days, 14 days, 28 days, and 56 days, respectively.

#### 2.3.2. Making Experimental Models

To simulate the surgical procedures of LASIK, the rabbit 's eyes received suction for different periods of time (20 seconds, 3 minutes) by negative pressure generator in different groups (IOP >65 mmHg, 1 kPa = 7.5 mmHg). PTK cutting corneal epithelium (diameter of cutting was 6.0 mm, depth of cutting was 50 *μ*m), according to −9.00 D standards, corneal stroma was scanned by laser (diameter of cutting was 6.0 mm, depth of cutting was 127 vm), the wavelength of laser was 193 nm, energy density: 125 mJ·cm^−2^, pulse frequency*：*10 HZ. Experimental control group were treated only by laser; the normal control group was not treated at all. 2.5 g·L^−1^ Chkoromycetinand eye drop was administrated to all rabbit's eyes in experimental groups after LASIK.

#### 2.3.3. Making Specimen

The sample (optic nerve and posterior retina) was extracted in postoperative instant, 7 days, 14 days and 28 days, respectively, in each group, take some of the sample (1 mm^3^), fixed (2.5% Glutaraldehyde Buffer Solution), dehydrated (Acetone), positioned under the microscope, EPON812 embed, made ultrathin slice, electron stained, made electron slice. The other, fixed (10% Formaldehyde), paraffin section (thickness was 5 *μ*m), Hematoxylin-Eosin stained, observed under the optical microscope.

#### 2.3.4. Examination Techniques

**F-ERG Examination**

0.2 mL/kg^−1^ Ketamine was injected to anaesthetize rabbits. 5 g·L^−1^ dicaine was dropped into conjunctive sac to superficially anesthetize. Darkness adaption 2 hours, set electrode. To observe the amplitude of ERG a-wave and b-wave as outcome measure, the average of right and left eyes (normal control,experimental control) was used as a data to process, the left eyes were took as experimental eyes in other groups.

**F-VEP Examination**

0.2 mL·kg^−1^ Ketamine was injected to anaesthetize rabbits. 5 g·L^−1^dicainewas dropped into conjunctive sac to superficially anesthetize. set electrode. To observe the amplitude of F-VEP P_1_ wave as outcome measure, the left eyes were took as experimental eyes.

### 2.4. Statistics Processing

The Paired design *t*-test of stata 7.0 statistical software was used to statistics process. There was statistical significance when *P* < .05.

## 3. Result

### 3.1. Histopathological Examination Results

**Figure 1:**
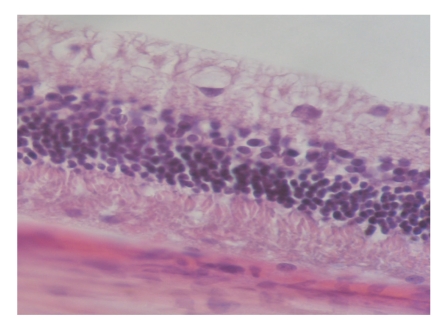
Shortly after suction 20 seconds, every layer of rabbit retina is in order (HE × 400).

**Figure 2:**
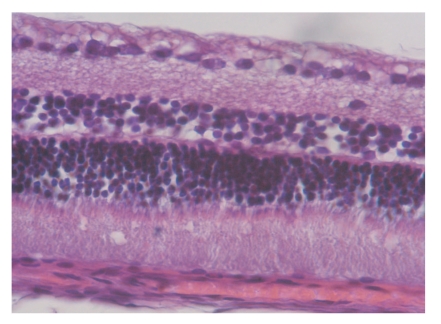
Shortly after suction 3 minutes, retinal nerve fiber layer and retinal ganglion cells changed sharply such as edema, every layer of rabbit retina is not in order (HE × 400).

The normal retinal structure of rabbit was divided into 10 layers from inside to outside. The number of external granular layer cells was the biggest, and the internal granular layer cells was smaller, and the gangliocytes was the smallest. All of the cells were in different size, round or oval, and most of the nuclear were deviation. There was no obvious change in retinal structure in experimental control and suction 20 seconds compare with normal control (Figure 1). Shortly after suction 3 minutes, retinal nerve fiber layer and retinal ganglion cells changed sharply such as edema, the nuclear of gangliocytes disrupted and dissolved; the number of inner nuclear layer cells was decline and turbulence, and the layers of visual cells were not in order and intact (Figure 2).

### 3.2. Transmission Electron Microscope Examination Results

**Figure 3:**
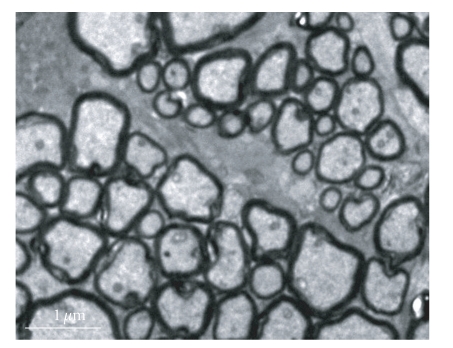
The normal fiber marrow scabbard of the optic nerve is tightness, no delamination (× 10 000).

**Figure 4:**
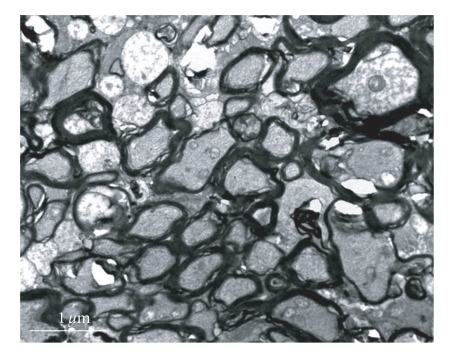
Shortly after suction 20 seconds, optic nerve fiber marrow scabbard is integrity, a little marrow scabbard evacuation, canaliculus and tiny threads are clear (× 10 000).

**Figure 5:**
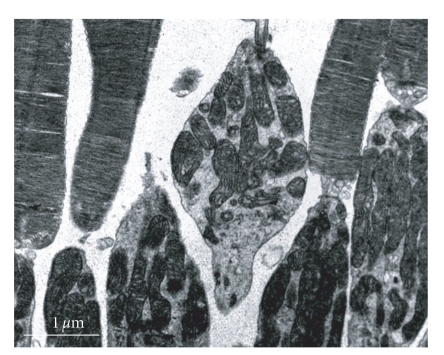
Shortly after suction 20 seconds, inner section cell member is integrity, the outer section is in order (× 10 000).

**Figure 6:**
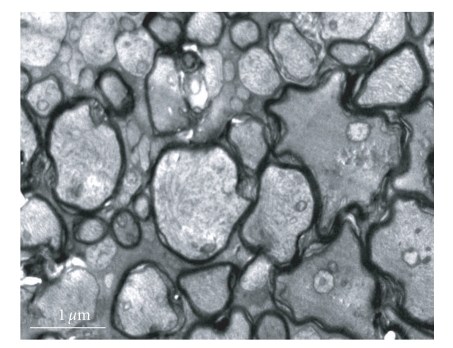
Shortly after suction 3 minutes, optic nerve fiber marrow scabbard is turbulence, canaliculus and tiny threads are no clear (× 10 000).

The normal fiber marrow scabbard of the optic nerve was tight, with no delamination; the electron-density was great, and canaliculus and tiny threads were clear (Figure 3). There was no obvious change in subcellular structure of retina and optic nerve in experimental control and suction 20 seconds compare with normal control group (Figures 4 and 5). Shortly after suction 3 minutes, optic nerve fiber marrow scabbard was turbulence, the electron-density was decline, canaliculus and tiny threads were not clear (Figure 6). Visual cell's cytoplasm of posterior polar retina was rupture, mitochondria swelled to go round, to sap cavity, and the outer section was edema and injury. Postoperative 7 days, optic nerve fiber marrow scabbard was in turbulence, and canaliculus and tiny threads were not clear. Inner section cell member was not in integrity, cytoplasm was in rupture and disorder, to sap cavity, canaliculus and tiny threads were not clear. The changes within postoperative 14 days were the same as those within 7 days. 

### 3.3. The Result of F-ERG in Normal Control and Experimental Control

There was no obvious change in ERG a-wave and ERG b-wave in normal control (a-wav: 43.75 ± 16.54 *μ*v) and experimental control groups (a-wave: 38.57 ± 14.39 *μ*v) (*P* > .05). There was obvious change in ERG b-wave in left (normal control: 92.25 ± 11.26 *μ*v, experimental control: 93.14 ± 8.14 *μ*v) and right eyes (normal control: 98.21 ± 10.14 *μ*v, experimental control: 95.68 ± 13.14 *μ*v) in normal control and experimental control group (*P* > .05).

### 3.4. The Result of F-ERG in Negative Suction 20 Seconds

There was no obvious change in ERG a-wave in different time after operation in suction 20 seconds and normal control (*P* > .05). There was no obvious change in ERG b-wave in different time after operation in suction 20 seconds and normal control (*P* > .05), except after suction 20 seconds and recovery 10 minutes (Tables 1 and 2).

### 3.5. The Result of F-ERG in Negative Suction 3 Minutes

There was no obvious change in ERG a-wave in different time after operation in suction 3 minutes and normal control group (*P* > .05). There was an obvious decrease in ERG b-wave after suction 3 minutes and recovery for 10 minutes, 20 minutes, 30 minutes compared with normal control group (*P* < .05); until recovery 1 hour ERG b-wave recovered to the normal level compared with normal control group (*P* > .05). There was obvious change in ERG b-wave within 1 hour–56 days after suction and after different duration of suction (*P* > .05). 

### 3.6. The Result of F-VEP

There was no obvious change in VEP p_1 _after different duration of suction in negative suction 20 seconds and negative suction 3 minutes compared with normal control group (Table 3).

## 4. Discussion

Generally, the transient IOP is over 65 mmHg during LASIK, and it lasts about 20 seconds when using automatic rotating knife. According to animal experiments [4], the IOP could reach 80–230 mmHg at the moment of negative pressure generator sucking during LASIK; it could even reach 140–360 mmHg when making corneal flap. The purpose of this study was to explore the safety time of negative suction, and the influence of transient and rapidly high IOP on the tissue of eyes during LASIK. 

The sharp rise in IOP, evoked by negative suction during LASIK, might lead to local ischemia on optic nerve and field loss [5, 6]. From this study, we had found that there was no change in sub-cellular structure of retina and optic nerve in normal control and experimental control group, which proved that laser therapy itself did not affect structure of retina. There was no obvious change in retina under histopathology and electron microscope at each time point of negative suction 20 seconds, so we can conjecture that it is safe to use automatic rotating knife during LASIK. However, after suction 3 minutes, retinal nerve fiber layer and retinal ganglion cells changed sharply, such as, edema, inner nuclear layer cells was not in order, and ultrastructure had change markedly too; those changes were more obvious at recovery 14 days so that the longer negative suction last, the more obvious changes of optic nerve fiber marrow, cone cells and rod cells. Those changes became more obvious with the negative suction prolonging. This result was the same as Adachi's [7]. However, it could recover to the normal level in 28 days after LASIK, which indicated that such change was reversible. The IOP going up sharply could injure the tissue of retina in negative suction 3 minutes; the main injury was in optic nerve fiber layer, retinal ganglion cell layer and inner nuclear layer. By using both the left and the right eyes of each rabbit as case-control study, the influence of individual difference in experimental control group can be eliminated. According to this study, we could see that there was no obvious change in ERG a-wave after different duration of suction in negative suction 20 seconds and negative suction 3 minutes. There was a transient increase in ERG b-wave after suction 20 seconds and recovery 10 minutes, then ERG b-wave started to decrease to the normal level after recovery 20 minutes (*P* > .05). There was no obvious change in ERG b-wave during 30 minutes-56 days after suction. The exceptionally transient increase in ERG b-wave might be a stress reaction that retinal cells respond to the high IOP in negative suction 20 seconds, and it could recover to the normal level after recovery 20 minutes. This result shows that negative suction was not organically injured, the high transient IOP produces an irritable change in ERG b-wave, but the influence is reversible. Therefore, the changes of histology and function were consistent. 

However, There was a decrease in ERG b-wave after suction 3 minutes and recovery 10 minutes, then it gradually increased; but it was still in low-level after recovery 30 minutes, and it recovered to the normal level (*P* > .05) 1 hour later. Although there was an obvious decrease in ERG b-wave after suction 3 minutes compared with after suction 20 seconds, it took longer time to recover to the normal level after suction 3 minutes than 20 seconds, but it triggered temporary and unorganical change in ERG b-wave, leading to irreversible changes of retinal function. We believed that the high IOP caused by negative suction during LASIK, might result in transient ischemia of retina that caused stimulant amino acid to release more [8], such as glutamic acid. The glutamic acid was combined with special receptor on the surface of cells to affect the function of Na-K-ATP enzyme, and finally the cells were injured. There is no clinical significance until the tissue injury, to certain degrees, shows the functional change. The Müller cells and bipolar cells appeared to be ingestion function obstacles, then the layers' tissue of retina were injured indirectly, which lead to the change in ERG b-wave. It was said that that change was caused by cellular reaction aroused by the drastic rise and drop in intraocular pressure that stimulated the spider cell in sieve plate and matrice extracellulaire. The result of ERG was similar to that in the report of Luna [9]. 

In this context, the transient changes in ERG b-wave and sub-cellular structure of optic nerve and retina cells were caused by the sharp increase in intraocular pressure during LASIK; moreover, amplitude and incubation period of ERG a-wave and VEP-P did not change significantly after different duration of suction. The longer the negative suction lasts, the greater the amplitude of ERG b-wave, and the more obvious the changes in tissue injury. However, optic nerve, retinal organization structure and ERG b-wave could recover to the normal level after certain restoration times, so this change was reversible. Therefore we believe that to cut down the negative suction time as much as possible, can ensure the security of LASIK.

## Figures and Tables

**Table 1:** Changes of ERG a-wave in suction 20 seconds, 3 minutes and normal control groups (*μ*v).

	Item								
Group	Recovery	Recovery	Recovery	Recovery	Recovery	Recovery	Recovery	Recovery	Recovery
	10 minutes	20 minutes	30 minutes	1 hour	1 day	7 days	14 days	28 days	56 days
Suction 20	35.69	41.33	33.23	38.67	29.03	36.23	35.11	31.88	32.58
seconds groups	± 8.66	± 22.69	± 10.18	± 8.79	± 13.04	± 8.76	± 13.43	± 14.34	± 15.75

Suction 3	38.47	37.34	36.69	36.98	30.75	35.03	37.03	32.31	33.88
minutes groups	± 7.89	± 8.39	± 8.96	± 11.51	± 7.97	± 11.54	± 9.86	± 12.34	± 12.34

Control	37.85	30.93	33.46	35.01	29.11	29.49	33.69	36.27	34.66
groups	± 12.39	± 14.02	± 11.45	± 13.22	± 15.47	± 13.51	± 10.01	± 8.99	± 9.38

**Table 2:** Changes of ERG b-wave in suction 20 seconds, 3 minutes, and normal control groups (*μ*v).

	Item								
Group	Recovery	Recovery	Recovery	Recovery	Recovery	Recovery	Recovery	Recovery	Recovery
	10 minutes	20 minutes	30 minutes	1 hour	1 day	7 days	14 days	28 days	56 days
Suction 20	112.01*	94.51*	95.81	100.21	97.16	90.34	96.1	94.12	98.47
seconds groups	± 11.08	± 14.43	± 12.30	± 10.48	± 14.27	± 14.30	± 17.55	± 15.41	± 10.22

Suction 3	55.89*	67.73*	80.21^∗#^	89.6^#^	92.6^#^	89.8^#^	99.61	96.35	101.33
minutes groups	± 9.46	± 16.42	± 14.23	± 7.10	± 11.01	± 15.64	± 18.52	± 10.32	± 15.64

Control	84.96	86.59	98.16	92.12	86.45	88.75	98.47	113.28	105.67
groups	± 13.48	± 15.25	± 12.56	± 21.01	± 18.89	± 12.33	± 10.22	± 13.48	± 20.06

Note: Compared with normal control group, *P* > .05, *P < .05; compared with suction 20 seconds group, ^#^ *P* < .05.

**Table 3:** Changes of F-VEP P_1_ in suction 20 seconds, 3 minutes and normal control groups (*μ*v).

	Item								
Group	Recovery	Recovery	Recovery	Recovery	Recovery	Recovery	Recovery	Recovery	Recovery
	10 minutes	20 minutes	30 minutes	1 hour	1 day	7 days	14 days	28 days	56 days
Suction 20	25.50	21.33	23.11	28.18	27.93	22.42	24.36	22.50	22.57
seconds groups	± 4.36	± 3.15	± 2.19	± 2.19	± 3.04	± 2.67	± 3.43	± 1.38	± 5.12

Suction 3	24.37	27.31	21.46	30.18	30.75	25.13	27.13	22.23	23.18
minutes groups	± 4.89	± 2.39	± 5.96	± 3.34	± 4.97	± 1.45	± 4.16	± 2.34	± 1.45

Control	21.18	26.93	25.46	28.01	29.11	23.42	23.34	26.17	24.64
groups	± 2.29	± 4.12	± 1.45	± 3.12	± 5.07	± 3.05	± 2.21	± 3.23	± 3.23
